# Optimization of the Parameters of a Minimal Coagulation Model

**DOI:** 10.3390/bioengineering12101111

**Published:** 2025-10-15

**Authors:** Carolin Link, Gábor Janiga, Dominique Thévenin

**Affiliations:** 1 Institute of Biochemical Engineering, University of Stuttgart, Allmandring 31, D-70569 Stuttgart, Germany; 2 Laboratory of Fluid Dynamics and Technical Flows, Otto von Guericke University Magdeburg, Universitätsplatz 2, D-39106 Magdeburg, Germany; thevenin@ovgu.de

**Keywords:** thrombosis, blood coagulation, coagulation model, optimization, genetic algorithm

## Abstract

The formation of a blood clot within a vessel can result in its complete blockage. This phenomenon, known as thrombosis, can have severe consequences. In contrary, thrombosis can be sometimes desirable. Intra-aneurysmal thrombosis is the primary objective of an endovascular treatment aimed at occluding the aneurysm sac. The proper modeling of the coagulation system is, therefore, important for the prediction, early recognition, and prevention of these tendencies. In silico investigations based on computational fluid dynamics (CFD) extended by thrombosis models provide a valuable tool for a detailed analysis. Minimal models are particularly useful for practical purposes to reduce computational efforts. This work proposes an approach to adapt the parameters of a minimal model to reproduce the behavior obtained with a comprehensive description of the coagulation cascade. The objective is to obtain the same thrombin generation curves while reducing strongly computational costs. For this purpose, machine learning—based here on an evolutionary algorithm—is used to optimize the obtained agreement. By adapting the reaction rate coefficients, a significant improvement can be achieved. The obtained results pave the way for future applications of the improved model in complex configurations such as for planning personalized interventions. Notably, the minimal model will be used for CFD in future studies to take advantage of its low computational cost.

## 1. Introduction

Thrombosis is associated with many cardiovascular diseases with a strong impact on morbidity [1,2,3]. A blood clot forms in an intact or in a diseased vessel by the activation of the coagulation system without proper physiological reason. Thrombosis has been shown to lead to ischemic heart disease, ischemic stroke, and venous thromboembolism [2]. Ischemic heart disease and stroke are among the 25 leading causes of death in the United States. Meanwhile, venous thromboembolism, which is classified as deep-vein thrombosis, pulmonary embolism, or both, affects 300,000 to 600,000 people annually only in the U.S. with a mortality of 20–30% [4]. In Europe, 38,929 pulmonary embolism-related deaths were counted annually between 2013 and 2015 [3]. Even though the probability of dying from cardiovascular diseases has decreased slightly in recent years [1,3], the burden of this disease is still particularly high. Additionally, the growing tendency of obesity will increase again the risk for thrombosis and related diseases. On the other hand, thrombosis can also be a desired effect, for instance, inside an intracranial aneurysm sack after endovascular treatment (see [5]).

A better understanding of the coagulation system is fundamental for prediction, diagnostics, and treatment but also for the regulation of thrombosis. The balance between procoagulant and anticoagulant factors prevents thrombotic tendencies and provides protective mechanisms in case of vessel damage. At the same time, better control of blood coagulation and, therefore, of intra-aneurysmal thrombosis could lead to new therapies with the help of thrombosis in the future. Nowadays, several detailed as well as reduced in silico models exist, describing blood coagulation with a focus on the different aspects of blood coagulation, for example coagulation kinetics, clot formation, or hemodynamics (e.g., [6,7,8,9,10,11,12,13,14,15,16]).

The underlying studies often neglect the full biochemical reaction pathway controlling thrombus formation. Sometimes, they even apply only indirect indicators, like residence time or shear rate. Li et al. [17] investigated whether the distribution of nitric oxide might be a superior indicator of thrombosis compared to the commonly used hemodynamic parameters derived from wall shear stress. Li et al. [18] suggested a mathematical model to assess platelet activation and thrombosis within ventricular-assist devices. They applied three transport equations to model resting platelets, activated platelets and coagulation factors considering them as dilution chemicals passively transported with blood flow. Komiya et al. [19] conducted a comprehensive review of various thrombus formation methods—considering both arterial thrombi and venous thrombi—and pointed out the need for clinically viable approaches that can accurately predict thrombus formation and identify the risk of incomplete clotting already at the level of treatment planning.

Revealing further details of the coagulation system is important for a better understanding of the underlying process. However, for practical applications, such as ventricular-assist devices (VADs), needed to support proper cardiac function [18], minimal models with acceptable computational efforts are absolutely necessary. Another example for which reduced models are necessary is the planning of personalized interventions [8]. Minimal models are easier to handle, require less computational effort, and are usually less stiff from a numerical point of view, enabling larger timesteps, and a time integration relying on simple and fast explicit time-stepping algorithms. At the same time, the results still need to be accurate for the given conditions and as close as possible to the results of the detailed models. Ideally, the accuracy of a detailed model should still be reached with the computational cost of a minimal model—i.e., reduced by at least an order of magnitude.

For instance, Wagenvoord et al. [9] obtained thrombin generation (TG) curves experimentally and fitted the parameters of a minimal model to those with help of a pre-defined W-function with four parameters:

(1) $$\mathrm{TH}=W\left(a,b,c,t_{0},t\right)=abc\cdot e^{-bc(t-t_{0})}\cdot {\left(e^{b(t-t_{0})}-1\right)}^{\left(c-1\right)},$$

where TH is the thrombin concentration, *t* is the time, and $t_{0},a,b,\mathrm{and}c$ are the unknown model constants [9]. One finding is that different parameter sets are able to represent the experimental data. This means that different minimal mechanisms leading to a similar accuracy are in principle possible.

Ngoepe and Ventikos [8] further reduced the minimal coagulation model suggested by Wagenvoord et al., leading to what is called the reduced Wagenvoord model in the following; they then compared it with a much more detailed model by [6] (called the detailed model or Hockin model in the rest of the article, and widely accepted as a reference model) in a two-dimensional simulation to reveal similarities and differences. They claimed that both TG curves show an overall similar course with comparable lag times: an increase, a peak, and a following decrease phase. Still, it was observed that the Hockin model has a higher maximal thrombin concentration that is reached earlier than the peak of the Wagenvoord model and returns faster to a constant value. The fit was obtained using only a single set of parameters.

Going one step further, the present study aims at finding optimal parameters for a minimal coagulation model. The reduced model by Wagenvoord et al. is used as starting platform for an optimization algorithm combined with numerical simulations in order to fit as accurately as possible the reference, the detailed model by Hockin et al.

The optimization of kinetic constants has been considered in many studies for a broad variety of applications, from combustion all the way to blood coagulation. Classical methods for the optimization of reaction rate coefficients are trial-and-error approaches, gradient-based methods, multiple shooting, sensitivity studies, pseudo-random algorithms, and solution mapping methods (see [20,21,22]). More recently, self-adaptive evolutionary algorithms were introduced. Generally, self-adaptive methods outperform classical gradient-based methods like multiple shooting [23]. These more powerful methods have been applied for the optimization of reaction rates in combustion, for example in [24,25]. In the work by Methling et al. [26], a linear transformation model is proposed to facilitate analysis and optimization of chemical processes and circumvent the need for genetic algorithms. The relation between input and output parameters of the kinetic process is simplified by linearization. Distances between characteristic points on the concentration time profile of the simulation and the ones of the original model are defined and minimized. For this linear transformation model, a rapid reduction is also possible. It was found that the model enables a significant reduction in the numerical costs after optimization. Based on this work, Methling et al. [27] applied the optimized model for the combustion of fuel mixtures involving synthetic gas and natural gas.

In the work by Tyurin and Khanin [28], the Lagrange multiplier technique with Gauss projection was used to determine kinetic constants for the extrinsic blood coagulation pathway, using minimal protein consumption as a criterion. The constants were determined in such a way that the optimal zymogen and procofactor concentrations coincided with the observed values. The authors started with a model combined from different references, and optimized three kinetic constants. At the end, good agreement was obtained with the experiments [28].

Wang et al. [29] used a genetic algorithm to develop a reduced model of the coagulation cascade. Starting from an already simplified kinetic description involving 19 species (compared to the reference model of Hockin used in the present study, with 34 species), they obtained a 10-species reduced-order model able to reproduce the kinetics of fibrinogenesis.

Unfortunately, most coagulation models have not been validated against clinical data as discussed by Chelle et al. [30]. In this work, different coagulation models were validated and optimized by comparison to experimental data using a genetic algorithm. The calibration was first performed on the whole population to obtain one unique set of parameters. However, the resulting agreement was poor. It was then found that a better estimation could be obtained by changing only three parameters, leading to a subject-specific calibration—at the cost of generality.

Perini et al. [31] presented an iterative framework to reduce reaction mechanisms using the information of an element flux analysis and optimized the reaction rate constants using an evolutionary algorithm. This method was employed for the simulation of an internal combustion engine. The optimization of operating conditions based on genetic algorithms combined to an efficient reduction in reaction mechanisms is also discussed in [32].

A two-level optimization was proposed by Hansen and Shadden [33] to generate minimal coagulation models, using the detailed Hockin model as a reference. In the outer optimization, the optimal species for the minimal model are selected; in the inner optimization, the reaction rates for this network are determined. In addition to thrombin generation, the development of the tissue factor (TF) and its influence on the network were also considered. Promising results were obtained, but the automation of the model reduction reduced the influence of deeper biological insights to a more data-driven approach. While researchers can benefit from a fast and easy reduction, biologically important species and rates might be excluded if they do not improve the specific optimization metric, reducing interpretability.

The work in the present study concentrates on the optimization of reaction rates, starting from an already existing minimal model used successfully in many previous studies, particularly regarding intracranial hemodynamics—the main focus of our group in follow-up studies [34]—explaining why it has been retained here. In order to go beyond previous publications, (1) the present work accounts for reversible reactions—often found in practical systems, (2) unphysical reactions are eliminated from the start, and (3) more realistic Michaelis–Menten kinetic rates are applied.

The present article is structured as follows. First, the selected models are introduced and discussed. Afterwards, advanced approaches used in the present study are presented. Finally, the optimization of the reaction rates—taking into account different TF concentrations—is presented, before drawing conclusions.

## 2. Materials and Methods

### 2.1. Detailed Coagulation Model by Hockin

The detailed coagulation model by [6,35] is a highly complex, state-of-the-art approach in blood coagulation modeling.

In the present investigation, this model is considered the gold standard. Its original form comprises 34 differential equations, 42 rate constants, and 34 species. It takes into account most processes contributing to coagulation. Still, some side reactions, like the inhibition by protein C, are neglected. For the present study, the model has been slightly modified following Ngoepe and Ventikos [8] by replacing the biochemical complexes involving antithrombin as a single “species” called INACT, which represents all inactive products. Furthermore, the reactions involving intermediate steps in the reaction scheme of Hockin et al. [6] are separated into individual reactions. Finally, the full reaction scheme considered to be the reference for the present study, involving 31 reactions and 30 species, as well as the associated initial concentrations and diffusion coefficients, is given in Table 1 and Table 2.

Hockin et al. compared the results of their model with empirical data taken from the report of Butenas et al. [36] with a starting concentration of F-VIIa-TF of $5\times 10^{-9}$ mM and a varying amount of prothrombin. Good agreement was observed. In particular, the maximal thrombin concentration could be reproduced with high accuracy. However, the increase at the beginning of coagulation (during the initiation phase, controlling lag-time) is not very well described. Different possible reasons are provided concerning this discrepancy in [6]. Still, this detailed model is widely accepted as reference in the scientific literature.

### 2.2. Reduced Wagenvoord Model

The minimal model describing TG in plasma by Wagenvoord et al. [9] was slightly modified by Ngoepe and Ventikos [8], arguing that not all reactions contribute significantly to the overall prediction. As a consequence, and based on a sensitivity analysis, the reactions without any significant influence were neglected. This modified model is used as starting minimal model in the present study. In contrast to the detailed model by Hockin, the reduced Wagenvoord model given in the work by Ngoepe and Ventikos consists of only 4 reactions and 8 species, with one single inhibition pathway. The corresponding reactions and initial parameters are given in Table 3. The initial concentrations and the diffusion coefficients are taken from the Hockin model [6] as given in Table 2.

This model describes the most important steps of initiation, growth, and inhibition. Factor X (denoted as X) is triggered by the TF, and thereby tenase (Xa-Va) forms. This is a complex between the activated factor X (Xa) and the activated factor V (Va). Tenase catalyzes the cleavage of prothrombin (PT) to thrombin (TH). The produced TH amplifies the process by catalyzing the production of more tenase. The last equation describes the inactivation of TH by AT. INACT denotes the inactive product of this reaction. This last reaction does not follow the Michaelis–Menten kinetics but is assumed to be a simple irreversible reaction. In their work, Wagenvoord et al. state that the influence of activated protein C and thrombomodulin could be simulated without introducing corresponding reactions [9]. Ngoepe and Ventikos also compared this model with data taken from the work of Butenas et al. [36] for varying surface-bound TF concentrations and showed that the outcomes are indeed comparable.

It must be stated that both models have been already used successfully in many past studies. Nevertheless, improving further the kinetic parameters by extensive comparisons with a comprehensive reference model would be very valuable to obtain an increased accuracy regarding in particular TG curves while saving time and computational power. This will open the door to simulations of realistic configurations based on CFD relying on this improved minimal model.

### 2.3. Optimization

In order to further improve the existing reduced model, the reaction rate coefficients of the Wagenvoord model are adapted in order to obtain the best possible agreement compared to the detailed coagulation model of Hockin [6]. In the existing literature, the proposed values exhibit significant variability and are associated with large uncertainties. Since the minimal model is only an approximation of true processes, it is not possible to measure directly the corresponding parameters in any experiments. For this reason, a fit based on an optimization procedure is a more reasonable solution.

For this purpose, the optimal values of all the model parameters are investigated using numerical simulations. The corresponding, zero-dimensional—i.e., purely time-dependent—computations rely on the in-house code ALBORZ [37,38], which is a hybrid code combining the lattice Boltzmann method (LBM) for hydrodynamics with a Finite-Difference (FD) solver for species transport equations. ALBORZ can efficiently handle single-phase flows, particulate flows, and—as needed for the present study—reacting flows as well. Even though no flow is considered yet in this article, we have decided to apply from the start our in-house code for the optimization, as the next objective is to simulate hemodynamics in combination with biochemical reactions, building on our experience regarding simulations of intracranial aneurysms and their treatment [34,39,40]. In this manner, it is ensured that the obtained, reduced coagulation model is directly integrated into the code and immediately available for future CFD studies. The improvement of the model parameters is obtained by a combination of simulations relying on ALBORZ with an optimization algorithm implemented in Python as detailed in what follows. The simulation results are saved every 500 timesteps, with each timestep equal to $5.0\times 10^{-3}$ s. In order to eliminate any possible numerical disturbance that could be introduced by the initialization of the computational setup, the first 20 data-points are excluded from the evaluation. No flow velocity is computed as part of the solution since this is a purely time-dependent (zero-dimensional) investigation. Therefore, only the FD part of the solver is active for the present study, with a second-order explicit time integration—the LBM solver being unused in the absence of any flow.

**Figure 1:**
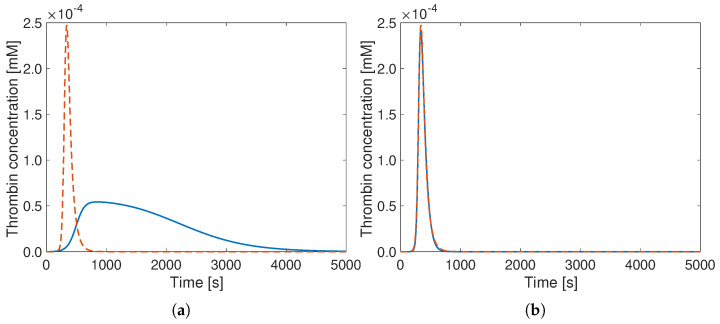
Thrombin concentration curves versus time for the detailed model by Hockin (**- -**) and the reduced Wagenvoord model (**–**) with (**a**) its default parameters from the literature and (**b**) the optimal reaction rate constants obtained by the procedure described previously (values from Table 5). Here and in all further figures, the results are shown for a total physical duration of 5000 s, though the optimization procedure considers only the first 625 s.

The central purpose of the present optimization is to obtain the best possible thrombin generation (TG) curves. Therefore, the square root of the sum of squared distances (SSSD) between each point of the TG curves—as shown in Figure 1—is used as an objective function, to be minimized:

(2) $$\mathrm{SSSD}=\sqrt{\sum_{1}^{N_{data}}{\left({\mathrm{TH}}_{W}-{\mathrm{TH}}_{H}\right)}^{2}},$$

where $N_{data}$ is the number of evaluated simulation points—the same for all cases—and ${\mathrm{TH}}_{W}$ and ${\mathrm{TH}}_{H}$ are the thrombin concentrations at the evaluated points for the reduced model (based originally on the reduced Wagenvoord model) and the detailed model by Hockin (taken as a reference), respectively. The successful minimization of this objective function corresponds to the best agreement between thrombin concentration curves.

The optimization is performed with genetic algorithms [41] using the very efficient NSGA-II method [42]. Such algorithms mimic the ideas of biological reproduction. Children (also called offspring) are computed using the genes of their parents and introducing crossover and random mutations. All properties can then be evaluated in parallel. Only the fittest individuals—i.e., those with the smallest objective function values (smallest SSSD values)—survive and make it to the next generation. These robust algorithms provide globally optimal values and reduce the risk of getting stuck in a local optimum. For the present study, Python 3.6.5 and the pymoo package by J. Blank and K. Deb are used [43]. All the variables are coded as real values. For the selection process, a binary tournament is used. The genetic algorithm is finally continued for 300 generations as explained later. After initial tests, each solution contains a population size of 100 (meaning 100 different parameter sets for the reduced model). For the crossover operator, SBX (simulated binary crossover) is retained with a distribution index of $\eta_{c}=15$ and a probability of $p_{c}=0.9$. The mutation operator is activated with a distribution index of $\eta_{m}=20$ [43].

Acceptable ranges must first be selected as meaningful limits of the model parameters before starting the optimization. Due to the uncertainty of the model parameters and—more generally—of all biological processes, variations by two orders of magnitude compared to the original (minimum and maximum) values (listed in Table 3) are allowed. Though this would be possible, it was decided at first not to extend the acceptable bounds much further. The purpose is to obtain optimized parameters not too far away from the nominal values, which are based on extensive prior research and have plausible physiological meanings. This should ensure that the parameter set remains consistent with fundamental biophysical constraints, preventing numerically optimal but physiologically fully unrealistic values. Widening the bounds might have led to parameter estimates beyond any realistic ranges, reducing the biological relevance and interpretability of the model outcomes. As discussed previously, it was already observed in the original study by Wagenvoord et al. [9] that very different parameter sets can lead to a similar accuracy. Our hypothesis is, therefore, that it is possible to obtain an optimized set of parameters for which the different values still remain within physiologically meaningful bounds.

The seven design variables considered during the optimization (i.e., all reaction constants of the reduced model) are listed in Table 4 together with the corresponding ranges used finally in the optimization. As can be seen in this table, while the range between lower and upper limit indeed covers at least two orders of magnitude in both directions (as just explained), it is necessary to extend slightly these bounds for some variables. The reason for that is that the original procedure delivered some optimal parameters located exactly at the limit of the initially defined parameter space. Since this reveals potential for further improvement, the corresponding bounds are extended further slightly to avoid this issue. The finally prescribed bounds for all parameters are shown in Table 4. With those values, all optimal parameters are found sufficiently far from the boundaries to ensure optimality.

As mentioned above, the SSSD between the predictions of the reduced and the reference model is used as single objective function—to be minimized.

For the optimization, the obtained curves are compared only for the first 625 s of the physical process (excluding the first 20 data-points as explained before), always including the peak obtained with the detailed model by Hockin. Further tests involving even longer physical times (up to 5000 s) show that concentrating on this initial phase is sufficient and leads to the same results while accelerating the procedure.

This is a single-objective optimization, so a best solution is obtained in each generation. This best individual is systematically selected for monitoring the optimization process, guided by its objective function (SSSD, steadily decreasing). The NSGA-II algorithm demonstrates once again its ability to consistently enhance the objective function of the best individual. Tracking the progress of the optimization, it is observed that the progress became very slow, with only a negligible reduction in SSSD values after 285 generations. For this reason, the optimization is stopped after 300 generations.

After finding optimal parameters, the robustness of the obtained results is further investigated. For this purpose, the concentrations of the other species (beside TH) obtained with the reduced Wagenvoord model (and optimal parameters) are compared with those of the Hockin model, used further as a reference. Furthermore, the initial TF concentration are varied to investigate if the optimal parameters are still valid for different values. Therefore, the range from 1 to 25 pm TF (the same as originally used in Hockin et al. [6]), corresponding to physiological conditions, is considered. Finally, the initial concentration of all factors found in a normal blood flow are multiplied (by 0.25, 0.5, 2, and 4) compared to their default value in order to describe the fact that, even in a healthy population, such differences by a factor of 4 in both directions are naturally present [44]. It is, therefore, important to check that the reduced model with optimal parameters still provide stable and acceptable predictions within this range.

## 3. Results

The optimal model parameters for the reduced Wagenvoord model (using the Hockin model as reference) are identified using first the single-objective optimization described in the previous section. The algorithm is pursued for 300 generations with a population size of 100 (i.e., considering 100 different parameter sets in each generation). This large database, with a total of 30,000 parameter combinations to be assessed, is found necessary to obtain accurate results—but leads, of course, to high computational costs. Overall, the entire optimization process is completed within 10 days of wall-clock time on a standard desktop Intel PC with 3.30 GHz and 5 computing cores. This is fully acceptable in the present study since the process is carried out only once. In the case that the optimization procedure is repeated many times, perhaps in the frame of a later patient-specific implementation, the wall-clock time could be easily reduced by orders of magnitude using a high-performance parallel computer and evaluating the different individuals in a population simultaneously.

This procedure delivers the optimal reaction parameters that are shown in Table 5 together with the original values and the errors compared to the reference (i.e., SSSD-values) for the two sets of parameters. The optimal values for the model constants k_cat,1_, k_M,2_ and k_f,4_ are found roughly in the same range as their original values (changes by less than a factor 50). All other optimal parameters are found to be very different from the original values. All the optimal parameters are found well within the bounds of the finally defined search space for the optimization. The parameter with the largest modification compared to the original values is k_M,1_, increased by a factor of 5310.

The obtained improvement is considerable since the SSSD value is reduced by almost two orders or magnitude (last line of Table 5) thanks to the optimization. Visually, this can also be clearly seen in Figure 1, which shows the comparison of the TG curves for both the original (Figure 1a) and the optimal parameters (Figure 1b) for the same minimal model.

**Figure 2:**
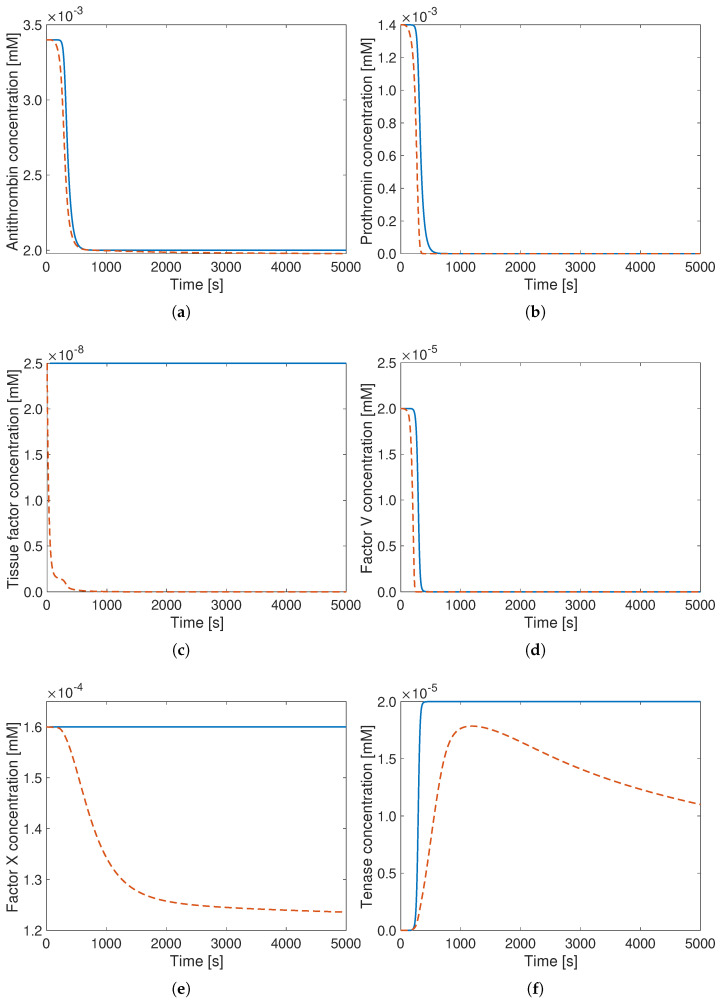
Concentration curves obtained with the (reference) Hockin model (**- -**) and with the reduced Wagenvoord model using optimal reaction rate constants (**–**). This figure shows the time evolution of the concentration of the species (**a**) AT, (**b**) PT, (**c**) TF, (**d**) V, (**e**) X, (**f**) XaVa. Note the different vertical scales.

Comparing now the concentration curves for all other species, shown in Figure 2, the curves for antithrombin (top left), prothrombin (top right), and factor V (middle right) demonstrate very good agreement between the reference (Hockin model, in orange) and reduced Wagenvoord model with optimized parameters (blue).

Since the TF just acts as a catalyst for the first reaction in the reduced Wagenvoord model, its concentration remains constant—as expected. Factor X and tenase do show some deviations from the curves of the reference (Hockin model), but still with the proper order of magnitude (note the different vertical scales). Due to the very strong model reduction, such deviations must be expected.

On the positive side, the computing time needed to reach 5000 s of physical time on the employed PC amounts to 155 s for the reduced model using optimal parameters, while the same simulation with the original Hockin model (reference) requires 2940 s of computing time. Therefore, the speed-up amounts to a factor of almost 20.

In order to check further the robustness of the optimal model, the initial concentrations of the species, such as factor X (X), factor V (V), prothrombin (PT), and antithrombin (AT) are changed systematically by multiplying those with factors varying between 0.25 and 4 around their standard value. All cases are simulated with the detailed model by Hockin (reference), the reduced Wagenvoord model with original parameters, and the reduced Wagenvoord model with optimal parameters, finally comparing the obtained TG curves (the most important target quantity).

**Figure 3:**
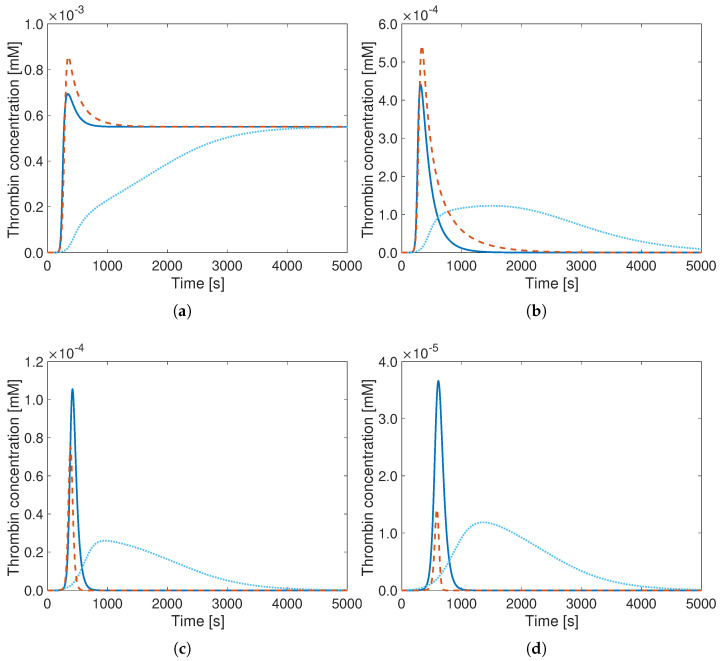
Thrombin generation curves of the Hockin model (**- -**), of the reduced Wagenvoord model with default parameters (**..**), and of the reduced Wagenvoord model with optimal parameters (**–**) for different initial AT-concentrations. The values for the initial AT-concentrations are (**a**) $0.85\times 10^{-3}$ mM, (**b**) $1.7\times 10^{-3}$ mM, (**c**) $6.8\times 10^{-3}$ mM, and (**d**) $13.6\times 10^{-3}$ mM AT which correspond to the 0.25-, 0.5-, 2-, and 4-fold of the standard initial concentration ($3.4\times 10^{-3}$ mM). Note the different vertical scales.

**Figure 4:**
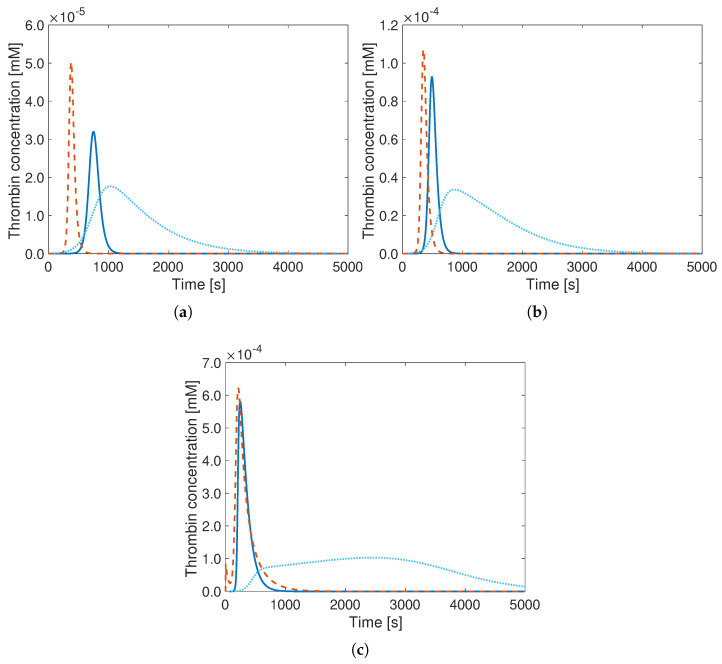
Thrombin generation curves of the Hockin model (**- -**), of the reduced Wagenvoord model with default parameters (**..**), and of the reduced Wagenvoord model with optimal parameters (**–**) for different initial PT-concentrations. The values for the initial PT-concentrations are (**a**) $0.35\times 10^{-3}$ mM, (**b**) $0.7\times 10^{-3}$ mM, (**c**) $2.8\times 10^{-3}$ mM, which correspond to the 0.25-, 0.5-, and 2-fold of the standard initial concentration ($1.4\times 10^{-3}$ mM). Note the different vertical scales. The (reference) Hockin model does not deliver stable results for a 4-fold increase in the initial concentration.

**Figure 5:**
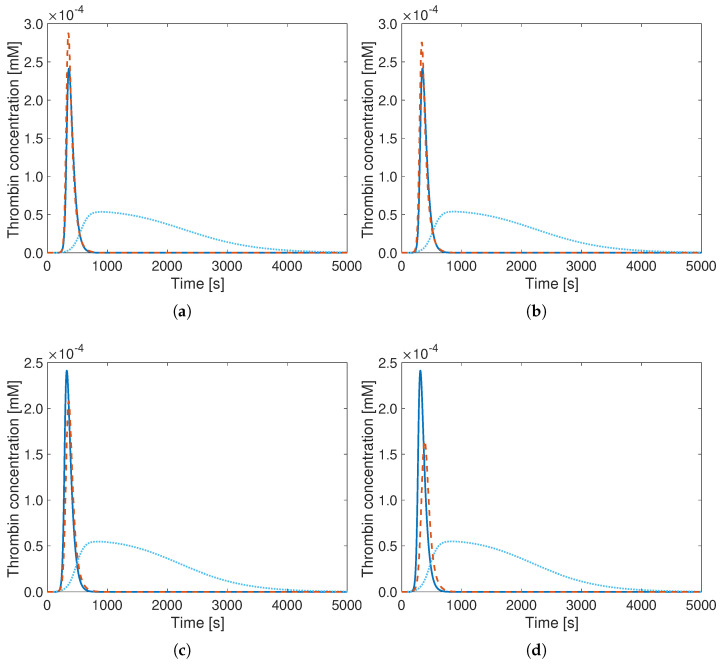
Thrombin generation curves of the Hockin model (**- -**), of the reduced Wagenvoord model with default parameters (**..**), and of the reduced Wagenvoord model with optimal parameters (**–**) for different initial X-concentrations. The values for the initial X-concentrations are (**a**) $0.4\times 10^{-4}$ mM, (**b**) $0.8\times 10^{-4}$ mM, (**c**) $3.2\times 10^{-4}$ mM, and (**d**) $6.4\times 10^{-4}$ mM X, which correspond to the 0.25-, 0.5-, 2-, and 4-fold of the standard initial concentration ($1.6\times 10^{-4}$ mM). Note the different vertical scales.

The results show that relevant changes in initial AT-concentrations can be well reproduced using the reduced Wagenvoord model with optimal parameters (solid blue lines) compared to the reference (orange). Going towards lower initial AT-concentrations, even initial AT values lower by a factor 4 still lead to a fair agreement (Figure 3). On the other hand, increasing the initial AT-concentration by a factor 4 leads to a noticeable discrepancy. However, the error is in all cases much smaller with the optimal parameters compared to the original ones, and the times at which the curves peak are still well reproduced. Similar observations can be drawn for PT (Figure 4), factor X (Figure 5), as well as for factor V.

**Figure 6:**
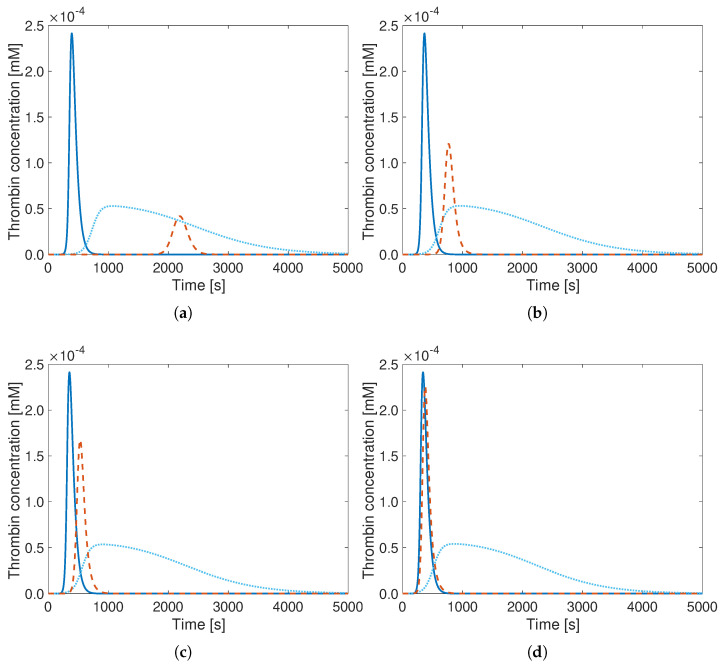
Thrombin generation curves of the Hockin model (**- -**), of the reduced Wagenvoord model with its default parameters (**..**), and of the reduced Wagenvoord model with optimal parameters (**–**) for different initial TF concentrations. The values for the initial TF concentrations are (**a**) $1.0\times 10^{-9}$ mM, (**b**) $5.0\times 10^{-9}$ mM, (**c**) $1.0\times 10^{-8}$ mM, and (**d**) $2.0\times 10^{-8}$ mM TF, which correspond to the physiological range.

From previous investigations, it is known that it is particularly difficult to properly reproduce the influence of the initial TF concentration with a minimal model. However, this initial TF concentration has indeed a large influence on the thrombin generation curve. A decrease in concentration results in a delayed initiation phase of TG [44], potentially challenging any accurate prediction relying on a minimal model, in which most coupling processes are strongly simplified. As can be seen in Figure 6, in which the results of the Hockin model (reference, in orange) and of the reduced Wagenvoord model with both standard (dashed blue) and optimal parameters (solid blue) are plotted, the reduced Wagenvoord model with standard model parameters is only very weakly sensitive to the initial TF value. Only the peak values of the curves vary slightly. On the other hand, the results of the reduced Wagenvoord model with optimal parameters vary noticeably as function of the initial value of TF. The predictions are much closer to the reference solution for initial values above $5.0\times 10^{-9}$ mM but show a large discrepancy for very small TF values (top left in Figure 6). In the simplified reaction mechanism following Wagenvoord, the TF concentration does not reduce with time (as it does for the Hockin model) since TF only acts as a catalyst of the first reaction. It is, therefore, extremely difficult to properly reproduce the evolution predicted by the reference model of Hockin. It can be hypothesized that TF-VIIa is formed more slowly such that the activation of X and IX is delayed. In addition, when a noticeable amount of thrombin is present, there is a positive feedback loop right at the beginning of the process, with an earlier activation of VII, activating in turn X [45]. Note that the better agreement observed for larger initial values of TF should be expected since the default initial value used in the Hockin model is $2.5\times 10^{-8}$ mM (Table 2), corresponding to the high range of physiological TF values.

Analyzing now all results simultaneously, it can be seen that the time at which the TG curves reach their peak—an essential modeling output—is systematically predicted more accurately using the optimal model parameters for all considered conditions, apart from the case with the lowest initial TF concentration (top left in Figure 6), far from the typical physiological conditions. The same applies to the peak values, highlighting the superiority of the optimal parameter set.

Different approaches have been considered in an effort to improve further the predictive performance for varying initial TF concentrations, particularly in the low range. The model structure was adapted and further regulations included (inactivation of TF by binding to factor VII, and/or inactivation by AT). However, none of these approaches led to any noticeable improvement. Directly taking three different initial TF concentrations into account in the optimization also did not improve the obtained agreement. Therefore, further progress is left for later studies.

## 4. Discussion

Minimal blood coagulation models that are able to describe accurately thrombin generation at a low computational cost are deeply needed for the further improvement of diagnosis, regulation, and therapy of thrombosis supported by CFD simulations. In this study, the reaction rate and Michaelis–Menten constants of the minimal reduced Wagenvoord model proposed by [9] and further modified by [8] were systematically optimized with the help of a genetic algorithm. The objective was to obtain the best possible evolution of the thrombin concentration curve by comparison with the comprehensive, state-of-the-art coagulation model by [6], used as a reference. The optimization led to a reduction in the error by almost two orders or magnitude. The TG curve obtained with the reduced Wagenvoord model using the optimal reaction parameters led to a much better agreement with the reference model for the reference case but also for a variety of initial concentrations of all relevant species. Therefore, the optimal parameter set appears to be robust.

Unfortunately, the influence of the initial TF concentration, which plays a crucial role for coagulation, could not be simulated perfectly with this approach. Several modifications have been tested, but none of them were successful. It is hypothesized that the minimal model intrinsically cannot describe properly the influence of TF and that it must be extended accordingly. In the meantime, the reduced model of Wagenvoord with optimal reaction parameters will be used in comprehensive investigations involving three-dimensional CFD simulations.

The same optimization framework could also in principle be used for adapting a model to patient-specific data instead of fitting a detailed coagulation model. In this manner, patient-specific parameters could be determined and later on used for precision medicine.

## 5. Limitations

To overcome the limitation discussed in the previous section (wrong prediction of the influence of initial TF concentration), modifications of the reaction scheme, as well as an optimization including more than one initial TF concentration have been considered.

One first idea was to include a reaction step in the minimal model that would be responsible for the inactivation of TF, hopefully improving the agreement between both models. As a first test, reaction 1 of the Hockin model (see Table 1) in which TF binds to FVII was included in the minimal model as a dead-end for TF.

The second possibility would be to include the inactivation of TF by AT. For this reaction, no default reaction rate constant is known. Therefore, the reaction rate constant of the inactivation of TF-VIIa with AT from the Hockin model (reaction 31, see Table 1) was assumed arbitrarily as a starting point for this parameter.

A third approach would be to add both the binding of TF to VII and the inactivation of this complex by AT. For all three possible modified models, the same optimization as described previously was undertaken. However, none of these modifications could lead to a noticeably better agreement for varying initial TF concentrations.

Another possible approach is to perform the optimization while taking into account more than one single initial TF concentration as performed in [33]. Following this idea, the simulation inside the optimization loop was performed for three different initial TF concentrations, and the objective function was averaged over the corresponding SSSD values. This was performed both for the original Wagenvoord model and for the third and last extensions of this model as described above. Once again, both tests did not lead to satisfactory results. This shows that an adaption of the existing Wagenvoord model apparently cannot agree with the results of the reference Hockin model when varying the initial TF concentration.

## 6. Conclusions

Minimal blood coagulation models that are able to describe accurately thrombin generation at a low computational cost are needed for the further improvement of diagnosis, regulation, and therapy of thrombosis supported by 3D CFD simulations. Several minimal models have been proposed in the literature. By optimizing the reaction rate constants, these models can hopefully be further improved and provide, as much as possible, the same results as much more complex and computationally expensive models.

In this study, the reaction rate constants of the minimal Wagenvoord model described in Wagenvoord et al. [9], further reduced by Ngoepe and Ventikos [8], is optimized with the help of a genetic algorithm. The objective is to obtain the same TG curve as the complex, state-of-the-art model by Hockin et al. [6], used as a reference. The optimization leads to a reduction in the deviation between both TG curves (from the Hockin model, and from the reduced Wagenvoord model) by almost two orders of magnitude, using a single-objective algorithm implemented in the Python package pymoo. The TG curve of the reduced model with optimal parameters shows in general much better agreement for different initial concentrations of the involved species, both regarding the peak time and the peak value. Unfortunately, the influence of the initial TF concentration cannot be simulated properly, particularly at low values. It is hypothesized that a proper adaption to different TF concentrations is not possible for this minimal model without changes in the reaction scheme, adding more degrees of freedom. This is the subject of future work.

In spite of this limitation regarding TF, the minimal model following Wagenvoord with optimized parameters will now be used for complex CFD studies since it improves prediction accuracy and at the same time leads to an acceleration of all computations by a factor of about 20, enabling much more complex investigations.

## Figures and Tables

**Table 1:** Reactions and rate constants of the modified Hockin model used as reference [6]. The dash (–) indicates that there is no backward reaction.

Step	Reaction Pathway	Forward Reaction Rate [mM^−1^s^−1^]	Backward Reaction Rate [s^−1^]
1	TF + VII $\overset{k_{+1}}{\underset{k_{-1}}{⇌}}$ TF–VII	3.20 $\times \;10^{3}$	$3.10\times 10^{-3}$
2	TF + VIIa $\overset{k_{+2}}{\underset{k_{-2}}{⇌}}$ TF–VIIa	$2.30\times 10^{4}$	$3.10\times 10^{-3}$
3	TF–VIIa + VII $\overset{k_{3}}{\to }$ TF–VIIa + VIIa	$4.40\times 10^{2}$	–
4	Xa + VII $\overset{k_{4}}{\to }$ Xa + VIIa	$1.30\times 10^{4}$	–
5	TH + VII $\overset{k_{5}}{\to }$ TH + VIIa	23	–
6	TF–VIIa + X $\overset{k_{+6}}{\underset{k_{-6}}{⇌}}$ TF–VIIa–X	$2.50\times 10^{4}$	1.05
7	TF–VIIa–X $\overset{k_{7}}{\to }$ TF–VIIa–Xa	6 [mM]	–
8	TF–VIIa + Xa $\overset{k_{+8}}{\underset{k_{-8}}{⇌}}$ TF–VIIa–Xa	$2.20\times 10^{4}$	19
9	TF–VIIa + IX $\overset{k_{+9}}{\underset{k_{-9}}{⇌}}$ TF–VIIa–IX	$1.0\times 10^{4}$	2.4
10	TF–VIIa–IX $\overset{k_{10}}{\to }$ TF–VIIa + IXa	1.8	–
11	Xa + PT $\overset{k_{11}}{\to }$ Xa + TH	7.5	–
12	TH + VIII $\overset{k_{12}}{\to }$ TH + VIIIa	$2.0\times 10^{4}$	–
13	VIIIa + IXa $\overset{k_{+13}}{\underset{k_{-13}}{⇌}}$ IXa–VIIIa	$1.0\times 10^{4}$	$5.0\times 10^{-3}$
14	IXa–VIIIa + X $\overset{k_{+14}}{\underset{k_{-14}}{⇌}}$ IXa–VIIIa–X	$1.0\times 10^{5}$	$1.0\times 10^{-3}$
15	IXa–VIIIa–X $\overset{k_{15}}{\to }$ IXa–VIIIa + Xa	8.20	–
16	VIIIa $\overset{k_{+16}}{\underset{k_{-16}}{⇌}}$ VIIIa_1_L + VIIIa_2_	$6.0\times 10^{-3}$ [s^−1^]	22 [mM^−1^]
17	IXa–VIIIa–X $\overset{k_{17}}{\to }$ VIIIa_1_L + VIIIa_2_ + X + IXa	$1.0\times 10^{-3}$ [s^−1^]	–
18	IXa–VIIIa $\overset{k_{18}}{\to }$ VIIIa_1_L + VIIIa + IXa	$1.0\times 10^{-3}$ [mM]	–
19	TH + V $\overset{k_{19}}{\to }$ TH + Va	$2.0\times 10^{4}$	–
20	Xa + Va $\overset{k_{+20}}{\underset{k_{-20}}{⇌}}$ Xa–Va	$4.0\times 10^{5}$	0.2
21	Xa–Va + PT $\overset{k_{+21}}{\underset{k_{-21}}{⇌}}$ Xa–Va–PT	$1.0\times 10^{5}$	103
22	Xa–Va–PT $\overset{k_{22}}{\to }$ Xa–Va + mTH	63.5 [mM]	–
23	mTH + Xa–Va $\overset{k_{23}}{\to }$ TH + Xa–Va	$1.50\times 10^{4}$	–
24	Xa + TFPI $\overset{k_{+24}}{\underset{k_{-24}}{⇌}}$ Xa–TFPI	$9.0\times 10^{2}$	$3.60\times 10^{-4}$
25	TF–VIIa–Xa + TFPI $\overset{k_{+25}}{\underset{k_{-25}}{⇌}}$ TF–VIIa–Xa–TFPI	$3.20\times 10^{5}$	$1.10\times 10^{-4}$
26	TF–VIIa + Xa–TFPI $\overset{k_{+26}}{\underset{k_{-26}}{⇌}}$ TF–VIIa–Xa–TFPI	$5.0\times 10^{4}$	–
27	Xa + AT $\overset{k_{27}}{\to }$ INACT	1.5	–
28	mTH + AT $\overset{k_{28}}{\to }$ INACT	7.1	–
29	IXa + AT $\overset{k_{29}}{\to }$ INACT	0.49	–
30	TH + AT $\overset{k_{30}}{\to }$ INACT	7.1	–
31	TF–VIIa + AT $\overset{k_{31}}{\to }$ INACT	0.23	–

The lowercase “a” after a factor’s name represents the activated status of it. Factor names combined with “–” indicate chemical complexes of these factors. TF—tissue factor; VII—factor VII; X—factor X; TH—thrombin; IX—factor IX; PT—prothrombin; VIII—factor VIII; VIIIa_1_L—dissociated factor VIII, first domain; VIIIa_2_—dissociated factor VIII, second domain; V—factor V; mTH—meizothrombin; TFPI—tissue factor pathway inhibitor; AT—antithrombin; INACT—inactive product.

**Table 2:** Initial conditions for the Hockin model [6].

Species	Initial Concentration [mM]	Diffusion Coefficients [m^2^s^−1^]
TF	$2.5\times 10^{-8}$	$1.0\times 10^{-11}$
TH	0	$6.7\times 10^{-11}$
PT	$1.4\times 10^{-3}$	$5.0\times 10^{-11}$
FVII	$1\times 10^{-5}$	$5.8\times 10^{-11}$
FVIIa	$1\times 10^{-7}$	$5.8\times 10^{-11}$
TF-FVII	0	$1.0\times 10^{-11}$
TF-FVIIa	0	$1.0\times 10^{-11}$
TF-FVIIa-FXa	0	$1.0\times 10^{-11}$
TF-FVIIa-FXa-TFPI	0	$1.0\times 10^{-11}$
TF-FVIIa-FX	0	$1.0\times 10^{-11}$
FX	$1.6\times 10^{-4}$	$5.5\times 10^{-11}$
FXa	0	$6.2\times 10^{-11}$
TFPI	$2.5\times 10^{-6}$	$6.5\times 10^{-11}$
FXa-TFPI	0	$4.5\times 10^{-11}$
FIX	$9.0\times 10^{-5}$	$5.5\times 10^{-11}$
FIXa	0	$6.2\times 10^{-11}$
FVIII	$7.0\times 10^{-7}$	$4.4\times 10^{-12}$
FVIIIa	0	$3.5\times 10^{-11}$
FVIIIa1L	0	$3.5\times 10^{-11}$
FVIIIa2	0	$3.5\times 10^{-11}$
TF-FVIIa-FIX	0	$1.0\times 10^{-11}$
FIXa-FVIIIa	0	$3.2\times 10^{-11}$
FIXa-FVIIIa-FX	0	$2.9\times 10^{-11}$
FV	$2.0\times 10^{-5}$	$2.7\times 10^{-11}$
FVa	0	$3.7\times 10^{-11}$
FXa-FVa	0	$3.3\times 10^{-11}$
mTH	0	$6.7\times 10^{-11}$
AT	$3.4\times 10^{-3}$	$5.5\times 10^{-11}$
INACT	0	$4.5\times 10^{-11}$
FXa-FVa-PT	0	$1.0\times 10^{-11}$

The lowercase “a” after a factor’s name represents the activated status of it. TF—tissue factor; TH—thrombin; PT—prothrombin; FVII—factor VII; FX—factor X; TFPI—tissue factor pathway inhibitor; FIX—factor IX; FVIII—factor VIII; FVIIIa1L—dissociated factor VIII, first domain; FVIIIa2—dissociated factor VIII, second domain; FV—factor V; mTH—meizothrombin; AT—antithrombin; INACT—inactive product.

**Table 3:** Reactions and rate coefficients for the reduced Wagenvoord model adapted from [8].

Reaction Pathway	Forward Reaction Rate [${\mathrm{mM}}^{-1}$$s^{-1}$]	k_cat_ [s^−1^]	K_M_ [mM]
X + TF → Xa-Va + TF		$5.00\times 10^{-2}$	$1.00\times 10^{-4}$
PT + Xa-Va → TH + Xa-Va		$8.00\times 10^{-2}$	$1.00\times 10^{-3}$
V + TH → TH + Xa-Va		$2.50\times 10^{+1}$	$5.50\times 10^{-2}$
TH + AT → INACT	$5.00\times 10^{0}$		

K_M_ represents the Michaelis–Menten constant, and k_cat_ the catalytic constant, respectively. X—factor X; TF—tissue factor; Xa-Va—activated factor X binding to activated factor V (tenase); PT—prothrombin; V—factor V; TH—thrombin; AT—antithrombin; INACT—inactive product.

**Table 4:** Upper and lower limits for the parameters considered during the optimization. Variations by at least two orders of magnitude are allowed both in the increasing and in the decreasing directions. For some of the parameters, this range is later extended slightly in order to avoid optimal parameter values lying on the boundary of the accessible parameter space.

Reaction Pathway	Lower Limit	Upper Limit
k_cat,1_	$5.00\times 10^{-4}\;\left[s^{-1}\right]$	$5.00\times 10^{0}\;\left[s^{-1}\right]$
k_M,1_	$1.00\times 10^{-6}\;\left[\mathrm{mM}\right]$	$1.00\times 10^{+0}\;\left[\mathrm{mM}\right]$
k_cat,2_	$8.00\times 10^{-4}\;\left[s^{-1}\right]$	$8.00\times 10^{+1}\;\left[s^{-1}\right]$
k_M,2_	$1.00\times 10^{-5}\;\left[\mathrm{mM}\right]$	$1.00\times 10^{-1}\;\left[\mathrm{mM}\right]$
k_cat,3_	$2.50\times 10^{-1}\;\left[s^{-1}\right]$	$1.50\times 10^{+4}\;\left[s^{-1}\right]$
k_M,3_	$5.50\times 10^{-4}\;\left[\mathrm{mM}\right]$	$4.00\times 10^{+1}\;\left[\mathrm{mM}\right]$
k_f,4_	$5.00\times 10^{-2}\;\left[{\mathrm{mM}}^{-1}s^{-1}\right]$	$5.00\times 10^{+2}\;\left[{\mathrm{mM}}^{-1}s^{-1}\right]$

**Table 5:** Original and optimal rate constants for the reactions given in Table 3. The optimization is performed using a single objective genetic algorithm. The square root of the sum of squared distances (SSSD) is calculated using Equation (2). The optimal reaction rate constants identified by the procedure are rounded to 2 decimal places.

Reaction Rate Constant	Default Value	Improved Value
k_cat,1_	$5.00\times 10^{-2}\;\left[s^{-1}\right]$	$8.16\times 10^{-3}\;\left[s^{-1}\right]$
k_M,1_	$1.00\times 10^{-4}\;\left[\mathrm{mM}\right]$	$5.31\times 10^{-1}\;\left[\mathrm{mM}\right]$
k_cat,2_	$8.00\times 10^{-2}\;\left[s^{-1}\right]$	$4.55\times 10^{+1}\;\left[s^{-1}\right]$
k_M,2_	$1.00\times 10^{-3}\;\left[\mathrm{mM}\right]$	$4.77\times 10^{-2}\;\left[\mathrm{mM}\right]$
k_cat,3_	$2.50\times 10^{+1}\;\left[s^{-1}\right]$	$6.38\times 10^{+3}\;\left[s^{-1}\right]$
k_M,3_	$5.50\times 10^{-2}\;\left[\mathrm{mM}\right]$	$2.52\times 10^{+1}\;\left[\mathrm{mM}\right]$
k_f,4_	$5.00\times 10^{0}\;\left[{\mathrm{mM}}^{-1}s^{-1}\right]$	$1.56\times 10^{+1}\;\left[{\mathrm{mM}}^{-1}s^{-1}\right]$
SSSD (error)	$1.89\times 10^{-3}\;\left[\mathrm{mM}\right]$	$4.65\times 10^{-5}\;\left[\mathrm{mM}\right]$

## Data Availability

Data are contained within the article.

## Abbreviations

The following abbreviations are used in this manuscript:

ALBORZ	in-house hybrid code combining the lattice Boltzmann method with a finite-difference solver
CFD	computational fluid dynamics
INACT	inactive product
LBM	lattice Boltzmann method
PT	prothrombin
SSSD	sum of squared distances
TF	tissue factor
TG	thrombin generation
TH	thrombin
